# A Child With a Gastrocolic Fistula After Ingesting Magnets: An Unusual Complication

**DOI:** 10.7759/cureus.9336

**Published:** 2020-07-22

**Authors:** Alaa Ali, Saeed Alhindi

**Affiliations:** 1 Pediatric Surgery, Salmaniya Medical Complex, Manama, BHR

**Keywords:** magnets ingestion, foreign body ingestion, gastrocolic fistula, young child

## Abstract

Foreign body ingestion is frequently the cause of emergency visits in the pediatric population, and these cases are challenging to diagnose and manage. In particular, the ingestion of magnets is dangerous and can lead to serious complications and even death. Urgent endoscopic intervention or surgical exploration remains the best approach for removing multiple ingested magnets and preventing further injury to the gastrointestinal (GI) tract. We report a nine-year-old child with an adjustment disorder who developed a gastrocolic fistula following the deliberate ingestion of multiple magnets. The magnets were successfully retrieved after an emergency laparotomy, and the fistula was subsequently repaired.

## Introduction

In children, especially those aged six months to three years, foreign body ingestion is common and, in many cases, unintentional. Children can be attracted to a variety of foreign bodies such as coins, toys, jewelry, magnets, and batteries; this is mostly due to their appealing external surface, colors, and shapes. In cases where older children are involved, further medical attention is warranted because of the need to rule out underlying psychiatric or neurologic disorders [1,2].

The frequency of magnet ingestion is rising, an indicator of the growing number of toys manufactured with magnets incorporated in them [3]. Ingestion of magnets and button batteries can have significant morbidity and mortality [4]. For instance, intake of multiple magnets can lead to the entrapment of bowel loops between them, resulting in perforation, obstruction, peritonitis, bowel ischemia, and necrosis, as well as fistula formation [3].

## Case presentation

A nine-year-old girl presented to our accident and emergency unit with a three-day history of vomiting and complaints of abdominal pain. Her symptoms persisted despite receiving hydration and pain medications at a primary healthcare facility. The vomit was non-bilious and non-projectile, and contained no blood or mucus. Her oral intake was not affected; no dysphagia nor drooling was reported. She had no history of fever, upper respiratory tract symptoms, shortness of breath, chest pain, cyanosis, previous admissions, surgeries, or allergies. She did have a history of an adjustment disorder following her parents’ divorce three months earlier, but no history of developmental delay or intellectual disability.

On examination, she was fully alert, mildly dehydrated (dry mucous membranes), and complaining of epigastric pain without signs of respiratory distress. She was vitally stable, and her blood pressure was 105/60 mm Hg. Her heart rate was 80 beats/min. Her abdomen was soft and not distended, and she did not show signs of tenderness, guarding, or rigidity. The remainder of the examination was unremarkable.

**Figure 1:**
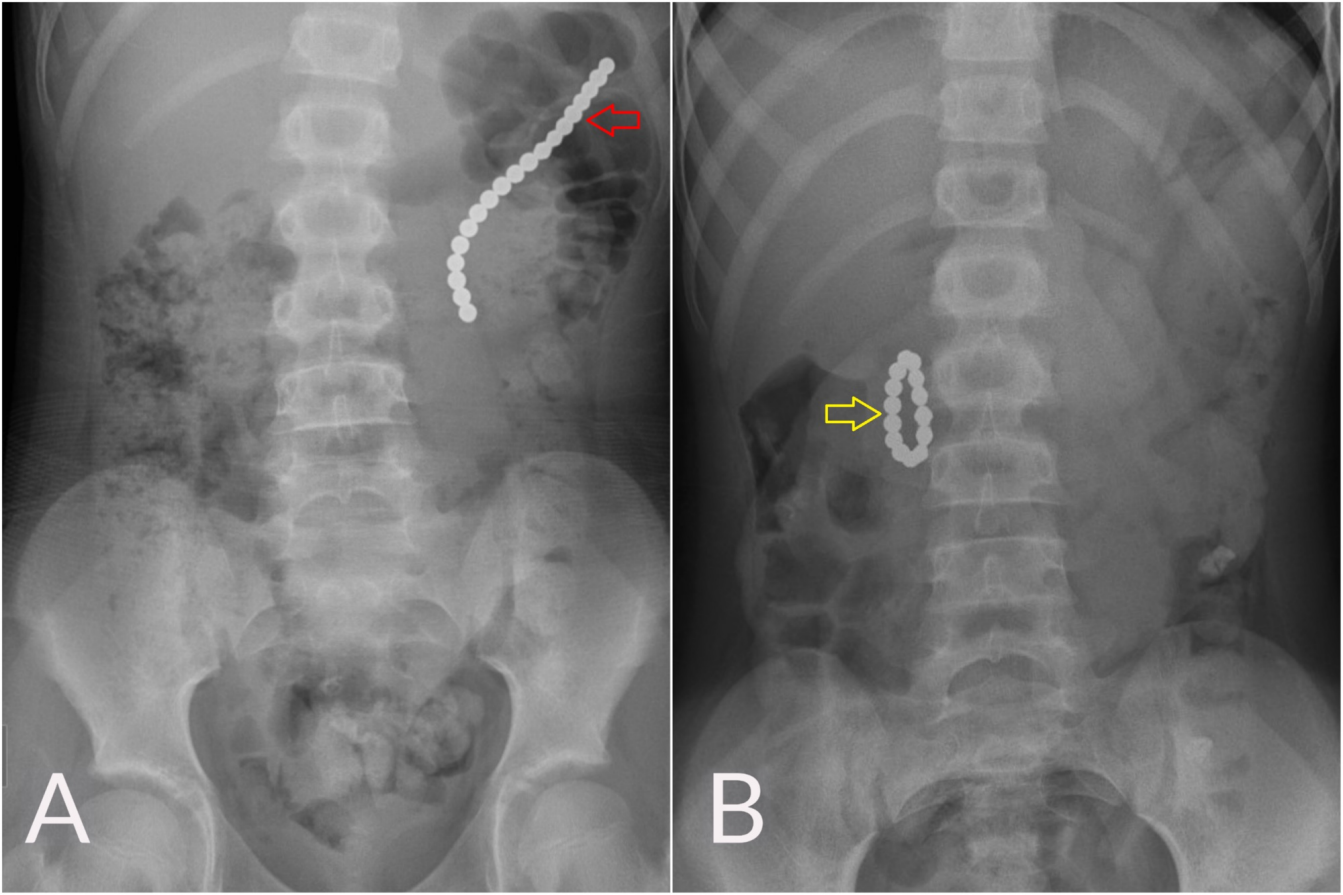
Abdominal x-rays (A) Abdominal x-ray shows a chain of 19 magnetic beads in the stomach (red arrow). (B) Intra-operative abdominal x-ray reveals that the 14 remaining magnets have migrated into the small bowel and formed a ring (yellow arrow)

An abdominal x-ray taken at admission showed a foreign body of metallic beads, about 10 cm in length, in the left upper quadrant of her abdomen (Figure 1A). Their ingestion was not witnessed, and the parents could not identify the object as seen on x-ray images. However, the patient later admitted to swallowing about 20 magnetic beads in succession, taken from a bracelet in the home. Although she acknowledged this was a wrong and harmful act, she refused to explain her action.

**Figure 2:**
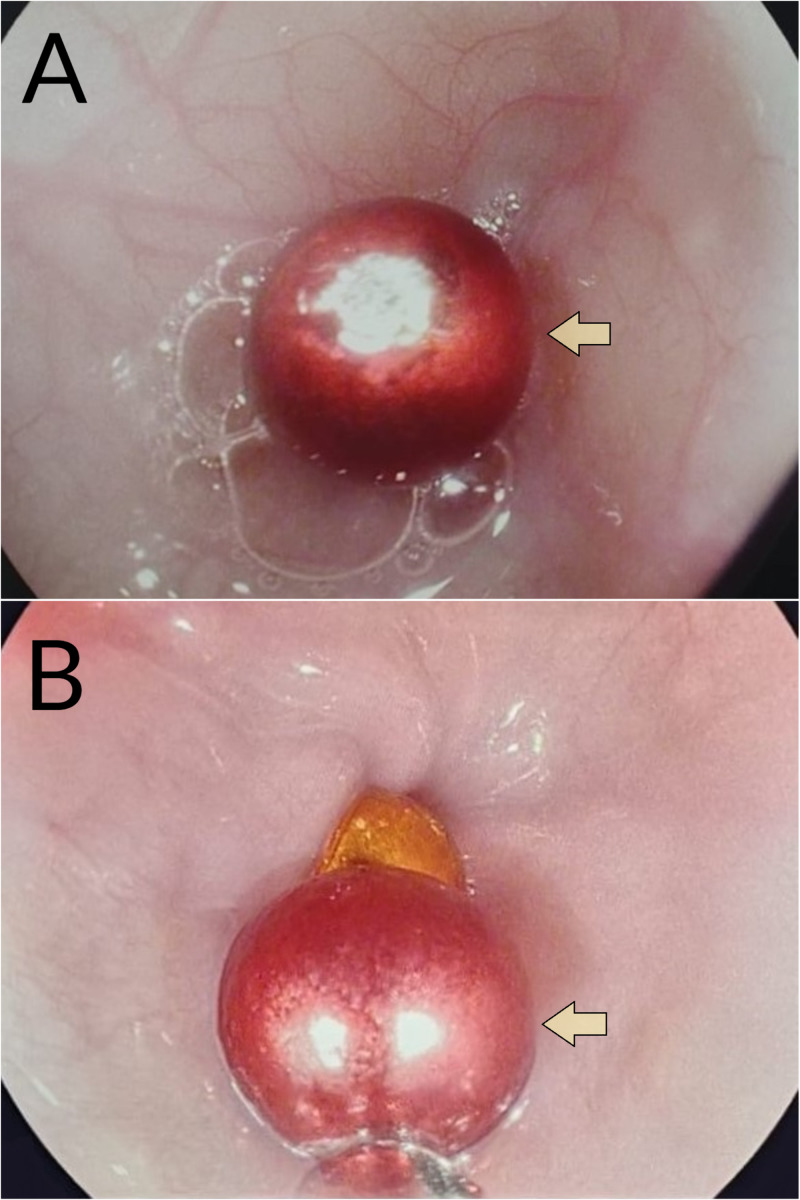
Endoscopy (stomach) (A) Endoscopic view of a magnetic bead within the stomach (yellow arrow). (B) Endoscopic view of a chain of at least two magnetic beads (yellow arrow) embedded within the gastric mucosa, and eroding into the anterior gastric wall

Given the location of the beads, an urgent upper GI endoscopy was performed during which multiple magnets were found embedded within the anterior wall of the stomach and surrounded by ulcerations (Figures 2A, 2B). Suspicion of GI perforation was high; therefore, the patient was scheduled for an emergency laparotomy. Her pre-operative laboratory tests included a complete blood count. Coagulation and biochemical profiles were within normal ranges.

**Figure 3:**
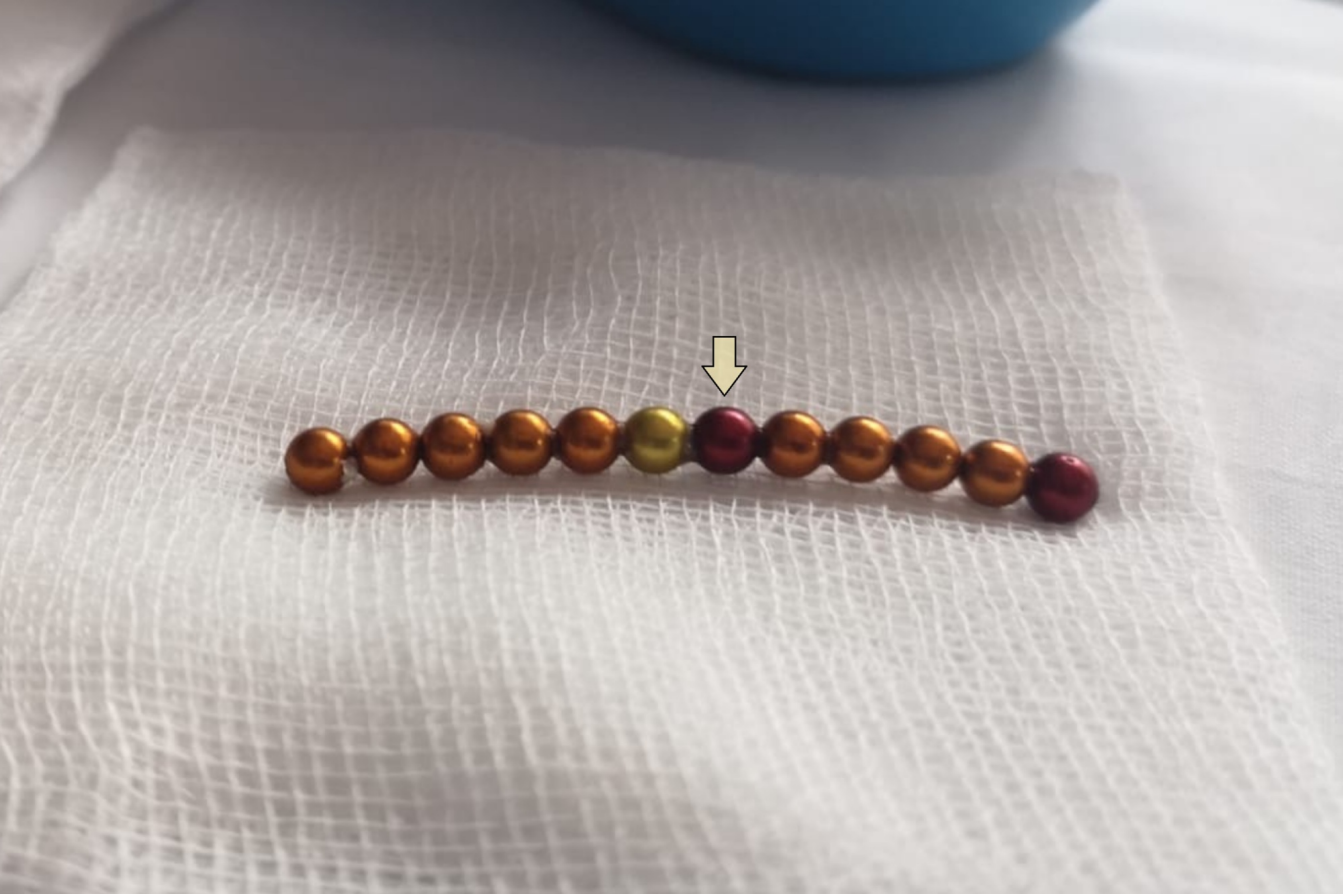
Part of the retrieved magnetic beads in a chain A chain of 12 of the 14 magnetic beads retrieved. It's composed of colorful rounded beads with each having a smooth surface, and measuring less than 5 mm in diameter (yellow arrow)

Intraoperatively, the greater curvature of the stomach was found adherent to the transverse colon, forming a gastrocolic fistula without intraperitoneal spillage. The magnets were present within the fistula. Only five beads were removed via the colonic opening after the fistula was divided. The remaining 14 were difficult to palpate, and fluoroscopy showed they had formed a ring and passed into the small bowel (Figure 1B). After recognizing and feeling the loop of magnets, an enterotomy was created, and the circular configuration of magnets was removed via this enterotomy. The magnetic beads were round in shape, each measuring about 4 mm in diameter (Figure 3). The fistula and enterotomy were repaired by primary closure.

The patient was clinically and vitally stable following surgery. The nasogastric tube was removed, and oral feeding began on the third postoperative day, bowel function returning the following day. On the fifth postoperative day, the patient was transferred to the child and adolescent psychiatric unit at our hospital for further psychiatric assessment and management of her adjustment disorder. She remained asymptomatic at a two-week follow-up appointment in the pediatric surgery clinic.

## Discussion

In the pediatric population, foreign body ingestion is a major health concern, given that the clinical scenarios are frequently challenging [2]. The estimated incidence is at least 100,000 cases annually, resulting in an annual mortality of approximately 1500 [1,5]. Younger children are especially susceptible because of their natural curiosity and oral mode of exploration. Most ingested objects are harmless when they are small and blunt, and they can be spontaneously evacuated from the GI tract. However, morbidity and mortality in these cases are influenced by several factors such as the type of the foreign body, its physical size, its shape, the presence of toxic components, and the site of lodgment within the GI tract [1].

Magnet ingestion, in particular, has increased over the last decade, largely the result of the prevalence of magnetic toys containing high-powered permanent magnets. Particular concern should be raised when ingested magnets are composed of iron, boron, and neodymium powder, which are 5 to 10 times stronger than simple iron magnets [4]. Swallowed magnets have a wide spectrum of clinical presentations that are most apparent within a week of ingestion; however, the presentation can be weeks to months later [6]. These cases can present with a sore throat, vomiting, constipation, abdominal pain, and even peritonitis [2]. Many single magnet ingestions are asymptomatic and do not require further intervention. In contrast, the ingestion of multiple magnets can have a complicated course with overt symptoms, even without evident peritoneal signs. Pressure necrosis with subsequent perforation, small bowel obstruction, volvulus, or fistula formation can result. Thus, at least half the patients who have ingested multiple magnets will require surgical intervention [3].

About 80% of the cases of foreign body ingestion can be diagnosed using plain radiography. X-rays can identify foreign bodies in most cases and can also determine the size, shape, and relationship between the foreign body and surrounding structures. However, plastic objects and most fish bones are radiolucent, and diagnosis can be challenging [7]. Magnetic beads can sometimes be mistaken for a single chain of beads or a bracelet, and the plurality of magnetic objects sometimes cannot be precisely determined using plain radiography or computed tomography [8,9]. In addition, intra-operative fluoroscopy is vital for confirming the complete extraction of foreign bodies and to localize them when they are difficult to palpate.

The management of ingested magnets is not standardized, and several protocols have been described in the literature. When a single magnet is less than 5 cm in size and has blunt edges, in the absence of other metallic objects or peritoneal irritation, observation is universally accepted [10]. However, when multiple magnets are ingested, several approaches have been proposed. When magnets are located within the prepyloric part of the GI tract, retrieval by endoscopy is recommended. In contrast, to prevent further damage to the bowel, surgical intervention by laparoscopy or laparotomy is advised when multiple magnets are found beyond the pylorus [6]. Tsai et al. reported five cases of multiple magnet ingestion, each presenting with non-bilious vomiting and abdominal pain [5]. All patients were initially evaluated using repeated x-rays followed by surgical exploration. Complications, including pressure necrosis, perforation, fistula formation, and bowel obstruction, occurred in four of these cases. In one, repeated imaging showed migration of the magnets to the bowel without symptoms or complications upon exploration, suggesting that asymptomatic children can be closely followed using serial x-rays to monitor the foreign body progression [5].

Early surgical consultation remains imperative when multiple magnets have been ingested, particularly when more than 12 hours have elapsed [11,12]. Nevertheless, endoscopic management of complicated cases has been described in the literature. Phen et al. reported a case of multiple magnet ingestion in a 19-month-old child complicated by a gastroduodenal fistula. It was successfully resolved by endoscopic removal and supportive care, avoiding the need for surgical intervention [11].

In our case, endoscopy was attempted but failed to allow extraction of all the magnets, necessitating surgical intervention for complete removal and correction of the gastrocolic fistula. Careful tissue handling during surgery and cautious retrieval of the magnetic objects is crucial to avoid creating multiple enterotomies, further morbidity, and lengthening the hospital stay, noting that magnets can easily migrate and adopt various shapes.

## Conclusions

Pediatric foreign body ingestion can present atypically and pose a diagnostic and management dilemma. Therefore, physicians should maintain a high index of suspicion in such cases, especially in older children for whom accidental ingestion is relatively unlikely. When multiple magnets have been ingested, early surgical intervention is crucial if endoscopy alone does not allow sufficient resolution, and a meticulous surgical technique is required for safe retrieval of the magnetic objects, thus preventing their further migration through the GI tract.
